# *In vitro* Assessment of the Impacts of Leaflet Design on the Hemodynamic Characteristics of ePTFE Pulmonary Prosthetic Valves

**DOI:** 10.3389/fbioe.2019.00477

**Published:** 2020-01-31

**Authors:** Guangyu Zhu, Yuan Wei, Qi Yuan, Li Cai, Masakazu Nakao, Joon Hock Yeo

**Affiliations:** ^1^School of Energy and Power Engineering, Xi’an Jiaotong University, Xi’an, China; ^2^NPU-UoG International Cooperative Lab for Computation and Application in Cardiology, Northwestern Polytechnical University, Xi’an, China; ^3^Cardiothoracic Surgery, KK Women’s and Children’s Hospital, Singapore, Singapore; ^4^School of Mechanical and Aerospace Engineering, Nanyang Technological University, Singapore, Singapore

**Keywords:** pulmonary prosthetic valve, pulmonary valve replacement, expanded-polytetrafluoroethylene, hemodynamic, *in vitro*

## Abstract

Prosthetic pulmonary valves are widely used in the management procedures of various congenital heart diseases, including the surgical pulmonary valve replacement (PVR) and right ventricular outflow tract reconstruction (RVOT). The discouraging long-term outcomes of standard prostheses, including homografts and bioprosthetic, constrained their indications. Recent developments in the expanded-polytetrafluoroethylene (ePTFE) pulmonary prosthetic valves provide promising alternatives. In this study, the hemodynamic characteristics of bileaflet and trileaflet ePTFE valve designs were experimentally evaluated. The *in vitro* tests were performed under the right ventricle (RV) flow conditions by using an *in vitro* RV circulatory system and particle image velocimetry (PIV). The leaflet kinetics, trans-valvular pressure gradients, effective orifice areas, regurgitant fractions, energy losses, velocity fields, and Reynolds shear stress (RSS) in both prostheses were evaluated. The opening of the bileaflet and trileaflet valve takes 0.060 and 0.088 s, respectively. The closing of the former takes 0.140 s, in contrast to 0.176 s of the latter. The trans-valvular pressure is 6.8 mmHg in the bileaflet valve vs. 7.9 mmHg in the trileaflet valve. The effective orifice area is 1.83 cm^2^ in the bileaflet valve and 1.72 cm^2^ in the trileaflet valve. The regurgitant fraction and energy loss of bileaflet are 7.13% and 82 mJ, which are 7.84% and 101.64 mJ in its bileaflet counterpart. The maximum RSS of 48.0 and 49.2 Pa occur at the systole peak in the bileaflet and trileaflet valve, respectively. A higher average RSS level is found in the bileaflet valve. The results from this preliminary study indicate that the current bileaflet prosthetic valve design is capable of providing a better overall hemodynamic performance than the trileaflet design.

## Introduction

Prosthetic pulmonary valves are widely used in the management procedures of various congenital heart diseases, including the surgical pulmonary valve replacement (PVR) and right ventricular outflow tract reconstruction (RVOT) (Fuller et al., 2019; Larsen et al., 2019). The reliability of the prosthetic valve has been a key to maintaining long-term right ventricular function after the treatments (Miyazaki et al., 2019).

The standard prosthetic pulmonary valves include homografts and bioprosthetic prostheses. The homografts have been used in PVR and RVOT for several decades. Although they are capable of providing superior hemodynamic performance, free of anticoagulation and encouraging early- to mid-term outcomes, the discouraging long-term durability and limited availability constrained their clinical application (Emani, 2012). Bioprosthetic valves, which are usually constructed from the decellularized bovine or porcine pericardium, overcome the shortage of homograft. However, the pulmonary bioprosthetic valves showed no significant improvement of long-term durability compared with the homografts, especially among young patients whose conduit size is smaller (Mitchell, 2016; Nomoto et al., 2016). For patients younger than 20 years old, the re-intervention rate of pulmonary prosthetic valve is 27% at 5 years (Chen et al., 2013; Dunne et al., 2016; Kwak et al., 2016; Mery et al., 2016; Nomoto et al., 2016) and sharply increased to 76% at around 15 years (Kwak et al., 2016; Mery et al., 2016). One of the most major causes of prosthesis dysfunction is calcification of the valve leaflets. Thus, the use of bioprostheses in young patients remains controversial.

Therefore, the search for an optimal pulmonary valve substitute is still ongoing. Among the newly proposed alternatives, the pulmonary valve prostheses made of the expanded-polytetrafluoroethylene (ePTFE) membrane have attracted great interest. The ePTFE is an inert material with microporous structure, which not only has good biocompatibility but also prevents the inflammation, calcification, and cell penetration that contribute to the valve deterioration (Zhu et al., 2015; Miyazaki et al., 2018; Sharifulin et al., 2018). Since Yamagishi et al., 1998 reported their early experiences (Yamagishi and Kurosawa, 1993), prosthetic pulmonary valves made of the ePTFE membrane have been widely used for PVR and RVOT in several centers in Japan and the United States. The follow-up results of the implantations of ePTFE prosthetic pulmonary valve have shown promising mid- to long-term outcomes. For the prostheses with trileaflet designs that mimic the native pulmonary valve configuration, the re-intervention rate at 5 years and 10 years was 7.7 and 23.9%, respectively (Brown et al., 2007; Miyazaki et al., 2018). In addition to the trileaflet design, the ePTFE valves with monoleaflet and bileaflet designs were also introduced to adapt the smaller pulmonary conduit size of pediatric patients as well as simplify the preparation procedures in the operation room (Yamagishi and Kurosawa, 1993; Quintessenza, 2014; Ootaki and Williams, 2018). Among these, the clinical satisfactory outcomes of the ePTFE prostheses with bileaflet design have been widely reported as well (Miyazaki et al., 2011; Lee et al., 2013; Mercer et al., 2018).

Despite the fact that the clinical outcomes of the ePTFE pulmonary prosthetic valves are encouraging, the impacts of prostheses design on the valvular hemodynamic performance is mostly unknown. In this study, the hemodynamic performances of trileaflet as well as bileaflet ePTFE valve prostheses designs were *in vitro* assessed under the pulmonary flow conditions.

## Materials and Methods

### Preparation of the Pulmonary Conduits

The pulmonary conduits were fabricated by casting of transparent silicon polymer (VTV, MCP-HEK Tooling GmbH, Kaarst, Germany) with a thickness and an annulus diameter of 3 and 25 mm, respectively. Both of the conduits have cast guidelines at the annulus level to guarantee the proper suture of leaflets, and the conduit for trileaflet prostheses included three sinuses that are adjacent to the annulus.

### Construction of the ePTFE Pulmonary Prosthetic Valves

The geometry of bileaflet prosthesis cusps was adopted from our previous design (Zhu et al., 2019), and the trileaflet cusps geometry was created based on the parameters from Thubrikar et al. (Thubrikar, 2018). Both of the bileaflet and trileaflet prostheses were designed to incorporate with conduits of 25 mm in diameter. The key geometrical parameters of the leaflets are listed in Table 1.

**TABLE 1:** Geometrical parameters of the ePTFE prosthetic valves.

**Design Parameters**	**Prosthesis Design**	
	**Bileaflet**	**Trileaflet**
*d*_b_ (mm)	25	25
*d*_c_ (mm)	25	25
*H* (mm)	30	21.6
*L*_f_ (mm)	36.6	29.2
*A* (mm^2^)	579.6	561.3

**d*_*b*_, the diameter of valve at base; *d*_*c*_, diameter of valve at base; *H*, overall valve height; *L*_*f*_, free-edge length; *A*, leaflet surface area.*

**FIGURE 1:**
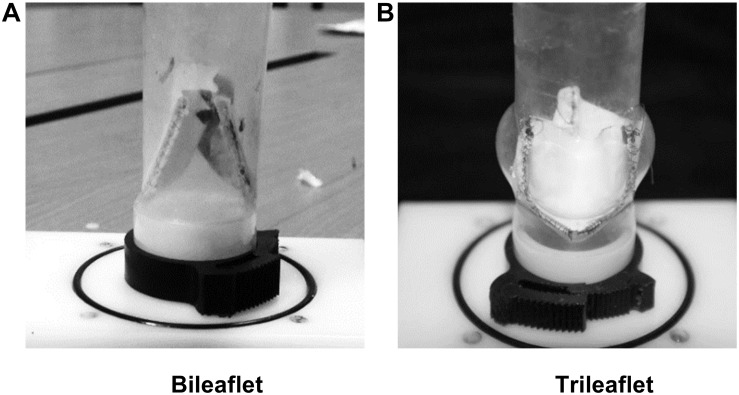
**(A)** Bileaflet and **(B)** trileaflet ePTFE pulmonary prosthetic valve models.

Based on the parameters, two sets of resin molds with geometry patterns that are identical to each design were 3D printed by using a stereolithographic 3D printer (Form 2, Formlabs Inc., Somerville, MA, United States). The ePTFE membrane of 0.1 mm thickness (Gore-Tex, Preclude Pericardial Membrane, W. L. Gore & Assoc., Flagstaff, AZ, United States) was placed in between the molds, and the membrane for trileaflet design was treated under 350°C to form the ePTFE into the desired valvular shape (Zhu et al., 2015). The leaflets of the prosthetic valves were prepared by trimming the membrane along the edge of the molds. After which, the commissures of the leaflets were sutured to the silicon conduits along with the guideline by using 4-0 polypropylene sutures. Figure 1 illustrates the constructed models of bileaflet and trileaflet ePTFE pulmonary prostheses for *in vitro* tests.

### *In vitro* Pulmonary Flow Loop

#### The Flow Loop

**FIGURE 2:**
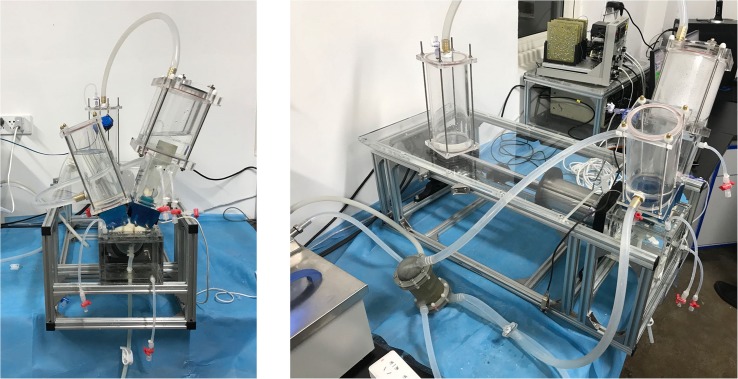
The *in vitro* right ventricular circulatory system.

To assess the hemodynamic performances of the pulmonary prostheses under physiological flow conditions, an *in vitro* RV circulatory system that is capable of mimicking the physiological pressure and flow rate waveforms of the right ventricle was designed and built (Figure 2).

The valved conduit to be assessed was mounted in the conduit chamber in between a silicon RV model and compliance chambers. The contraction and dilation of the silicon RV that generates the pulsatile flow profiles were passively driven by the programmable linear actuator. To control the pressure waveform, an adjustable resistive component was attached in the distal segment of the flow loop. At the tricuspid annulus site, a tilting disk mechanical heart valve (Björk-Shiley standard) was installed to serve as the tricuspid valve. In addition, a self-developed heat exchanger was connected to the tubing system to maintain the consistency of temperature during the experiments.

#### Flow Conditions

All tests were conducted at a stroke volume of 75 ml (5.4 L⋅min^–1^) and a heart rate of 72 beats⋅min^–1^. An aqueous solution of salt (0.9% by weight) and glycerol (42% by weight) was used as the blood analog. The dynamic viscosity and density of the working fluid is 3.52 mPa⋅s and 1038 kg⋅m^3^, respectively. Silver-coated hollow glass spheres of 10 [μ*m*] diameter were seeded into the fluid as the seeding particles for PIV. By controlling the air volume in the compliance chambers and resistive component, the peak systolic pressure, late diastolic pressure, and mean pressure of the pulmonary artery were set to 25, 10, and 16.8 mmHg, respectively. All the tests were performed at a constant temperature of 37 ± 1°C.

### Data Acquisition

To evaluate the hemodynamic performance of the prostheses, several parameters, including pulsatile flow profile, pressure profiles, internal velocity fields, and leaflet movements, are to be measured.

The pulsatile flow through the prosthetic valve was measured by an electromagnetic flowmeter (501D, Carolina Medical Electronics, East Bend, NC, United States); the flow probe was placed at the annular position that is 10 mm upstream the base of prosthetic valves. To monitor the systemic pressure as well as capture the trans-valvular pressure gradient, the pressure profiles of RV and pulmonary conduit were simultaneously measured at the pulmonary annulus level and 5 mm above the commissure level by using two pressure wires (SPC 330A, Millar Instruments, Inc., Houston, TX, United States). The flow and pressure signals were recorded at a sample rate of 1000 Hz by using NI 9201 modulus.

**FIGURE 3:**
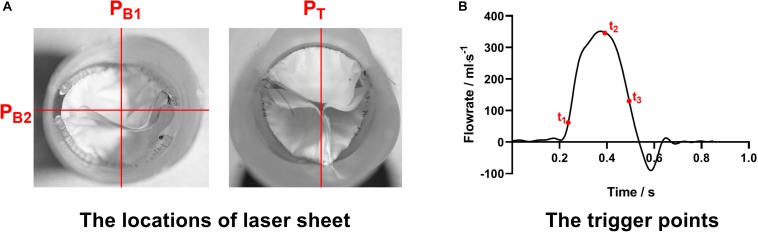
**(A)** The locations of laser sheets and **(B)** trigger points of the PIV measurement procedures.

The visualization of the inter-conduit flow fields was conducted by using a LaVision PIV system. The laser sheet of 532 nm wavelength was positioned at the two orthogonal center panels (P_B__1_ and P_B__2_) of the bileaflet prosthesis as well as at the center panel (P_*T*_) of the trileaflet prosthesis, respectively (Figure 3A). The cross-correlation image pairs in these panels were acquired at three different time points of the ejection phase (t_0_: acceleration, t_1_: peak and t_3_: deceleration) in a cardiac cycle (Figure 3B). In order to overcome the random errors that occurred during the measurement, at least 100 image pairs were taken in each measurement. The post-processing was conducted in the DaVis software, and the velocity fields in the abovementioned panels at each time point were obtained.

An endoscope that connected with a high-speed camera (FASTCAM-PCI R2 model 500, Photron United States, Inc., San Diego, CA, United States) was inserted into the distal end of the pulmonary conduit to record the motions of prosthetic valve leaflets. The leaflet motions over cardiac cycles were recorded at a frame rate of 250 fps.

All the sensors and devices were carefully calibrated prior to the measurements, and the data collections across the devices were synchronized by the transistor-transistor logic (TTL) signal from the controller of the linear actuator.

### Data Analysis

Based on the data acquired, the following hemodynamic parameters associated with the prosthetic valve complications were derived:

Regurgitant fraction (RF): the RF is a relative measure of volume overload, which is defined as the sum of regurgitant volume during valve closure (*V*_*R*_) and leakage volume (*V*_*L*_) over the forward flow volume (*V*_*F*_) during one cardiac cycle (ISO 5840, 2005. Cardiovascular implants. Cardiac valve prostheses) (Equation 1).

(1) $$\text{RF}=\frac{V_{R}+V_{L}}{V_{F}}\times 100\%$$

Effective orifice area (EOA): the EOA represents the minimal cross-sectional area of the flow jet downstream of the heart valves, and has been a standard clinical criterion of stenosis severity of valves (Garcia et al., 2004). Eq. 2 gives the definition of EOA:

(2) $$\text{EOA}=\frac{Q_{R\,M\,S}}{51.6\,\sqrt{\Delta \,P/\rho }}$$

where Δ*P* is the mean pressure gradient during the positive differential pressure period (mmHg), ρis the fluid density (g/cm^3^), and *Q*_*RMS*_ is the root mean square volumetric flow (ml/s) (Eq. 3); *t*_*s*_ and *t*_*e*_ denote the start and end of the period of positive differential pressure, respectively.

(3) $$Q_{R\,M\,S}=\sqrt{\frac{\int_{t_{s}}^{t_{e}}Q\,{\left(t\right)}^{2}\,d\,t}{t_{e}-t_{s}}}$$

Energy loss (*E*_*L*_): The *E*_*L*_ is the fluid energy that is consumed on the valve, which includes forward energy, closing energy, and leakage energy (Claiborne et al., 2013). The *E*_*L*_ at different cardiac phases could be estimated by using Eq. 4.

(4) $$E_{L}=0.1333\,\int_{t_{p\,1}}^{t_{p\,2}}\Delta \,p\,\left(t\right)\,Q\,\left(t\right)\,d\,t$$

where *t*_*p1*_ to *t*_*p2*_ is the period of different phases within a cardiac cycle.

RSS: The RSS is a term derived from the Reynolds decomposition of Navier–Stokes equations and indicates the turbulence level (Yousefi et al., 2017). Based on the velocity fields captured by PIV, the RSS fields in the immediate vicinity of the pulmonary valve prostheses could be calculated from Eq.5.

(5) $$\tau_{i\,j}=-\rho \,\bar{u^{\prime }\,v^{\prime }}\;$$

where τ_*ij*_ is the RSS, ρis the density of the fluid, and *u*′ and *v*′ are the fluid fluctuation in *x* and *y* directions.

## Results

### Leaflets Kinematics

**FIGURE 4:**
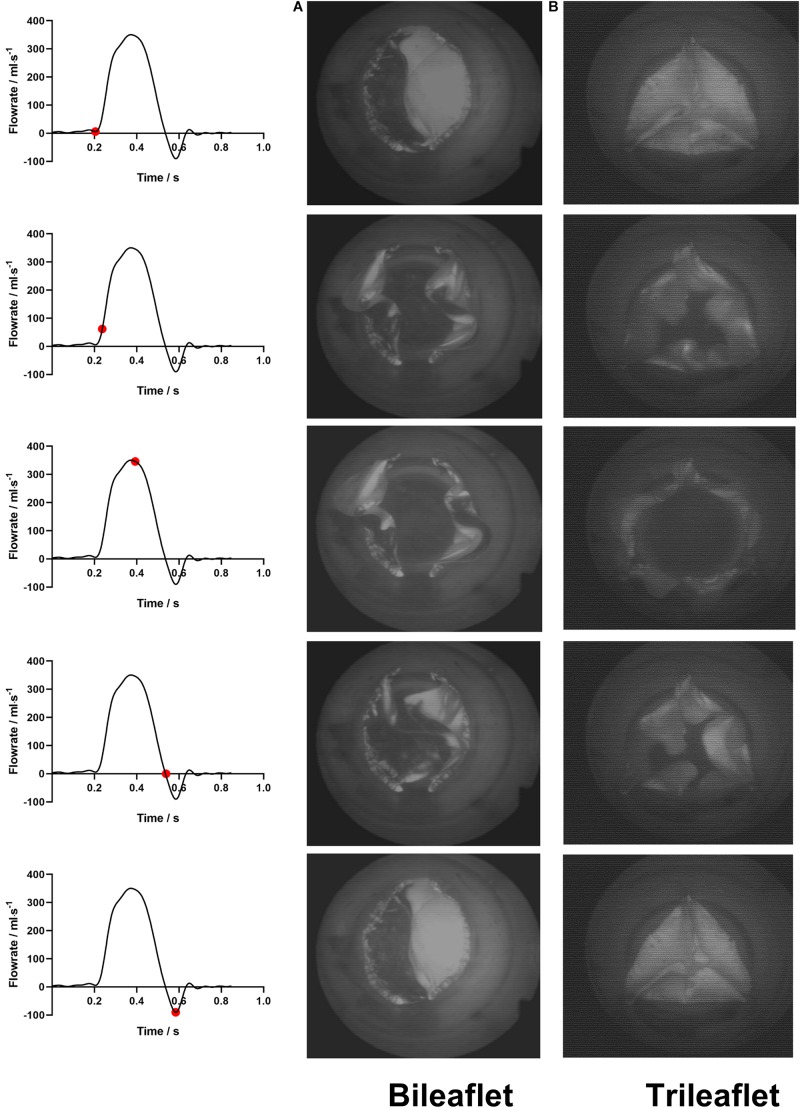
The leaflets motions of **(A)** bileaflet and **(B)** trileaflet prosthetic valves over a cardiac cycle.

The leaflet kinematics of both prostheses in a cardiac cycle were analyzed by comparing the frames from the high-speed camera records (Figure 4).

At the fully closed configuration, the coaptated leaflets of both valves twisted with each other counterclockwise, and the free edges formed S-shaped lines. The opening of the bileaflet valve started from the leaflet region in contact with the conduit first, and the center of leaflets separated from each other at the end of the opening phase. In contrast to the bileaflet prostheses, the opening of the trileaflet valve started from the center of the leaflets. The closing behavior of the valves was in a manner that is reverse to their opening.

Both of the prostheses presented large deformations as well as rapid motion of leaflets during the opening and closing. The duration of the opening phase, fully opened phase, and closing phase of the bileaflet and trileaflet valves is listed in Table 2. The results showed that the opening and closing periods of the trileaflet design under the RV flow conditions take 46.7 and 25.7% longer than its bileaflet counterpart. The duration of the entire open and close of bileaflet and trileaflet designs accounts for 38.07 and 45.30% of a cardiac cycle.

**TABLE 2:** Duration of the leaflets movement phases.

**Leaflets Motion Phases**	**Duration (s)**	
	**Bileaflet**	**Trileaflet**
Opening	0.060	0.088
Fully opened	0.116	0.112
Closing	0.140	0.176

#### Hemodynamic Characteristics and Flow Patterns

**FIGURE 5:**
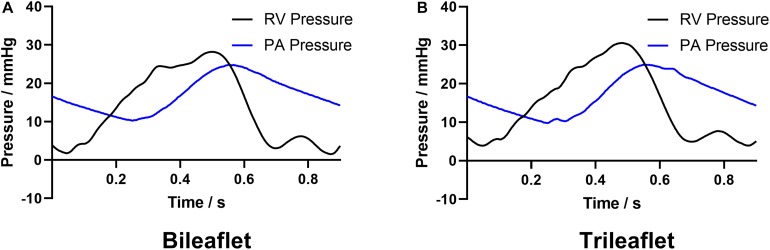
The acquired RV and PA pressure waveforms of **(A)** bileaflet and **(B)** trileaflet valves.

Figure 5 illustrates the pressure waveforms acquired from the different prosthesis designs. The peak systolic RV pressures of the bileaflet and trileaflet cases are 28.2 and 30.5 mmHg, respectively. Both valves showed an identical positive differential pressure period of 0.35 s. The mean pressure gradient in bileaflet prosthesis during this period is 6.8 mmHg, which is 13.9% lower than that in the trileaflet case.

**FIGURE 6:**
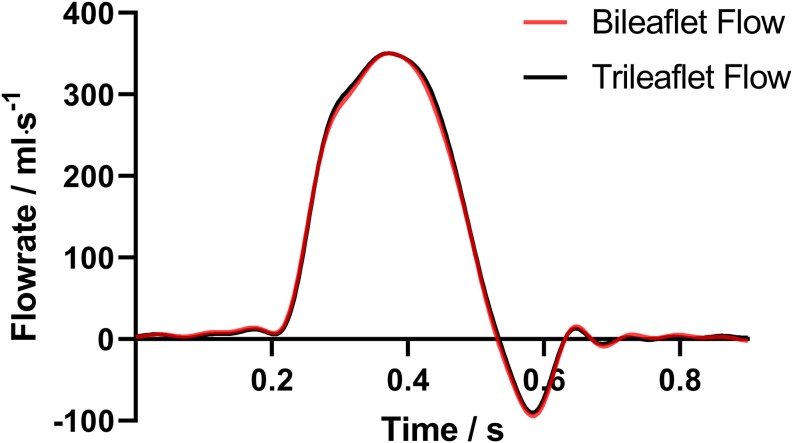
The acquired PA flow profiles of both valves.

The flow rate profiles are shown in Figure 6. The sum of the *V*_*R*_ and *V*_*L *_ in a cardiac cycle is 5.35 and 5.88 ml for bileaflet and trileaflet valves, respectively. Thus, the RF could be calculated by using Eq. 1, which was 7.13% for the bileaflet prosthesis and 7.84% for the trileaflet design.

In addition, the *Q*_*RMS*_, *E*_*L*_ and EOA of both prostheses were calculated, and the specific values of the parameters are listed in Table 3. Although the *Q*_*RMS*_ of both designs is approximately the same, the bileaflet design showed a 6.49% larger EOA as well as 19.32% smaller *E*_*L*_ than the trileaflet prosthesis.

**TABLE 3:** Comparison of the hemodynamic parameters.

**Parameters**	**Prosthetic Valve Designs**	
	**Bileaflet**	**Trileaflet**
*V*_*R*_ (ml⋅beat)	5.17	5.63
*V*_*L*_ (ml⋅beat)	0.18	0.25
RF (%)	7.13	7.84
*Q*_*RMS*_ (ml⋅s^–1^)	242.62	245.96
EOA (cm^2^)	1.83	1.72
E_*L*_ (mJ)	82	101.64

**FIGURE 7:**
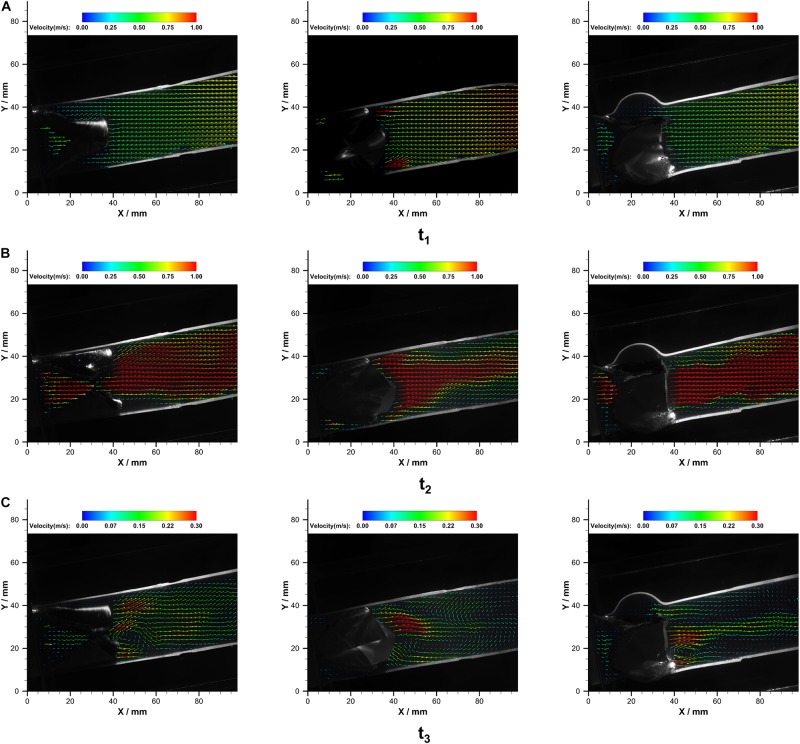
The velocity fields in P_B__1_ **(left)**, P_B__2_ **(middle)**, and P_*T*_ **(right)** at **(A)** t1, **(B)** t2, and **(C)** t3 over a cardiac cycle.

The velocity fields in the immediate vicinity of the valves at different cardiac phases are illustrated in Figure 7.

**FIGURE 8:**
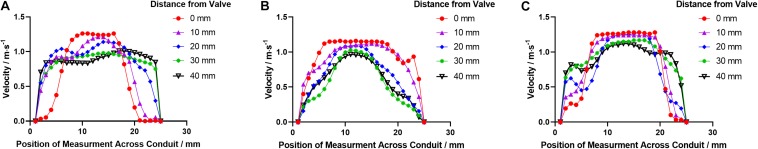
The velocity profile distributions downstream the leaflets in **(A)** P_B__1_, **(B)** P_B__2_, and **(C)** P_*T*_ at t_2_.

At the acceleration phase (*t*_1_), similar velocity fields that were uniformly distributed in the conduit were observed in P_B__1_ and P_*T*_. However, high-velocity jets near the conduit wall were observed in the P_B__2_. With the increase of flow rate, central jets gradually developed, and the maximum jet velocity in bileaflet and trileaflet cases reached 1.32 and 1.33 m⋅s^–1^ at the systolic peak, respectively. In the bileaflet prosthesis, the high-velocity jet immediately expanded after the valve, whereas it dominated the center in the trileaflet case. At the deceleration phase, a vortex that rotates clockwise near the leaflet tip was found in both cases. A higher overall velocity magnitude level presented in the bileaflet prostheses at the end systolic as well. In neither case did an obvious eccentric jet appear during the cardiac cycles. Figure 8 shows the velocity profiles in different cross-sections downstream of the prostheses at *t*_2_.

**FIGURE 9:**
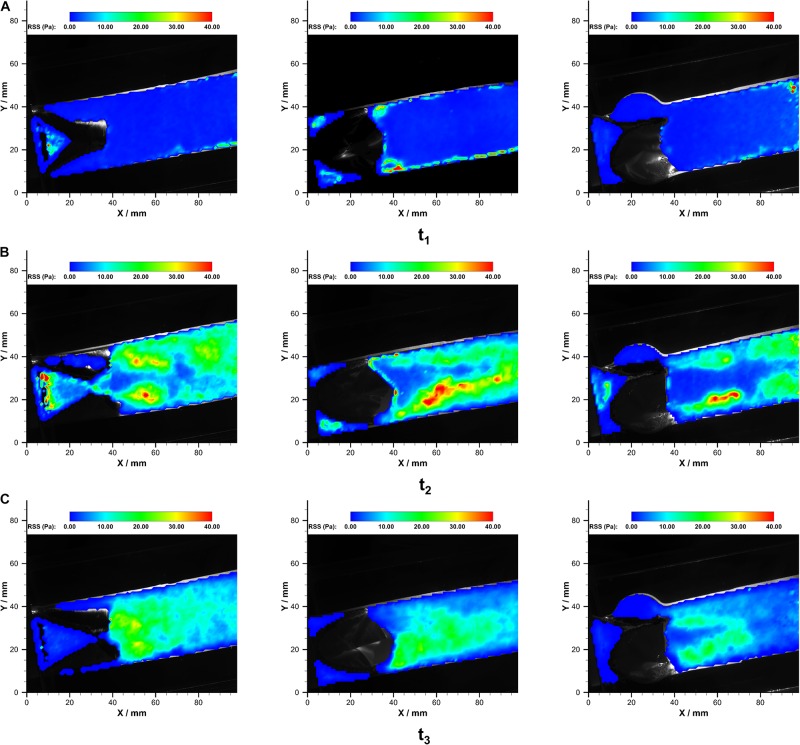
The RSS fields in P_B__1_ **(left)**, P_B__2_ **(middle)**, and P_*T*_ **(right)** at **(A)** t1, **(B)** t2, and **(C)** t3 over a cardiac cycle.

The RSS contours in both prostheses at the corresponding time points are illustrated in Figure 9.

**FIGURE 10:**
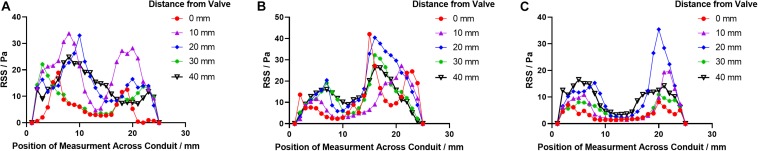
The RSS profile distributions downstream the leaflets in **(A)** P_B__1_, **(B)** P_B__2_, and **(C)** P_*T*_ at t_2_.

In both of the prostheses, streak-like high RSS regions appeared immediately near the leaflets due to the jet flow. The strength and distributions of RSS varied during the systolic phase. The maximum RSS at the systolic peak was 48.0 Pa and 49.2 Pa in bileaflet and trileaflet prostheses, respectively. In the bileaflet prosthesis, the symmetrical streak-like high RSS regions merged into a larger high RSS area that is approximately 10 mm from the tip of leaflets at the systolic peak. In the trileaflet prosthesis, by contrast, the high RSS strips remained separate at the efferent of the conduit. A low RSS region was found in the conduit center where the jet peak occurs. The RRS profiles in different cross-sections downstream the prostheses *t*_2_ are illustrated in Figure 10.

The specific values of averaged velocity and RSS at each cross-section are listed in Table 4.

**TABLE 4:** The values of averaged velocity and RSS.

**Distance from**	**Mean Velocity**			**Mean RSS**		
**Valve (mm)**	**(ml⋅s^–1^)**			**(Pa)**		
		
	***P*_B__1_**	***P*_B__2_**	***P*_*T*_**	***P*_B__1_**	***P*_B__2_**	***P*_*T*_**
0	0.65	0.92	0.80	5.13	11.21	3.35
10	0.74	0.84	0.83	17.50	9.87	6.14
20	0.85	0.65	0.77	14.04	16.53	10.90
30	0.84	0.53	0.91	8.96	12.35	5.10
40	0.86	0.57	0.86	13.40	12.52	9.01

## Discussion

In this study, the hemodynamic characteristics of bileaflet and trileaflet ePTFE pulmonary valve prostheses were experimentally investigated by using an *in vitro* pulmonary flow loop and PIV technique. To the best of our knowledge, this is the first *in vitro* investigation that reveals the impacts of the bileaflet and trileaflet ePTFE pulmonary prostheses designs on the hemodynamic characteristics.

### Impacts of Prosthetic Valve Design on Leaflet Kinematics

The results from the high-speed camera records showed that both prostheses investigated in this study are capable of providing normal function under RA flow conditions, including rapid opening and closing, no obvious obstruction at the fully open configuration, and sealed well after closing (Sacks and Yoganathan, 2007). Besides, the S-shaped lines at the closed position were observed in both cases. This phenomenon indicates that both valves are capable of providing abundant leaflet that guarantees a better coaptation of leaflets as well as provides a more safety closure (Xiong et al., 2010).

However, the geometrical differences of the valve leaflets resulted in several distinct leaflet dynamic features between the bileaflet and trileaflet valves. Firstly, the shorter opening and closing period of the bileaflet valve suggested superior leaflet flexibility to the trileaflet valve. The opening and closing period of the former take approximately 0.06 and 0.14 s, which is 0.088 and 0.176 s for the latter. Secondly, despite the leaflets of both valves being open in a symmetrical manner, the opening of the bileaflet valve started from the region that is in contact with the conduit instead of the leaflet center as the trileaflet valve. The large leaflet area of the bileaflet design could be the main reason for these phenomena. Although there is a lack of detailed data on the kinematics of ePTFE prostheses under RV conditions, the similar dynamic behavior of the bileaflet valve has been observed under aortic flow conditions as well (Zhu et al., 2019).

### Impacts of Prosthetic Valve Design on Hemodynamic Performance

The hemodynamic performance is another major concern in the evaluation of the pulmonary valve prostheses. Prosthesis with poorly hemodynamic performance is associated with several adverse events such as RV dilation, early deterioration of prosthetic valves, and decreased long-term survival rates (Rao et al., 2000; Tasca et al., 2015).

The RF is a critical parameter in the evaluation of reverse flow during valve closure. A valve with high RF would lead to the volume overload of the heart to provide adequate blood supply and result in the abnormalities of RV functions (Naeije and Badagliacca, 2017). In the clinical practices, the RF was graded as mild (RF < 20%), moderate (RF = 20%–40%) and severe (RF > 40%) (Mercer-Rosa et al., 2012). Although the RF of both valve designs that are investigated in this study is within the excellent level, the RF of bileaflet design is 9.06% lower than that of the trileaflet design. The faster close of leaflets and subsequently less regurgitant volume of bileaflet design could contribute to this phenomenon.

In the systolic phase, the evaluation of valve performance is mainly based on EOA. Despite the visual opening area of the bileaflet valve at the systolic peak being smaller than that of the trileaflet valve, the faster opening of the bileaflet valve during the acceleration phase benefits the decrease ofΔ*P*. Due to the *Q*_*RMS*_ of both valves being almost identical (difference less than 2%), the smaller Δ*P* of the bileaflet design contributed to its 6.40% larger EOA than that of the trileaflet design. Such a larger EOA of bileaflet design could potentially reduce the risk of prosthesis–patient mismatch in the PVR, which occurs when the EOA of the prosthesis is too small compared with patients’ body surface area (Zoghbi et al., 2009). This phenomenon is likely to be associated with the more flexible leaflets of bileaflet design, which is evident from the analysis of leaflet kinematics.

As a result of the larger EOA, the pressure gradient and energy loss in the bileaflet valve were found to be smaller than that of the bileaflet valve. In the quantitative comparison of the pressure gradient and energy loss across the valve, the bileaflet valve showed a 16% lower pressure gradient and 19.32 smaller energy loss than that of the trileaflet valve, respectively.

Though the direct quantitative comparison of the above hemodynamic parameters with those from other *in vitro* studies is limited by the lack of data acquired at corresponding conditions, the quantities of each parameter are still in line with their results (Nagy et al., 2000; Dur et al., 2010; Claiborne et al., 2013; Yamamoto et al., 2018; Pragt et al., 2019). Additionally, the superior performance of the same bileaflet valve design in terms of RF, EOA, pressure gradient, and *E*_*L*_ was also found under the aortic flow conditions (Zhu et al., 2019).

### Impacts of Prosthetic Valve Design on Flow Characteristics

In addition to the valve performance, the design of prosthetic valves affected the flow characteristics in the immediate vicinity of the leaflets as well.

#### Velocity Distributions

At the acceleration phase (*t*_1_), a distinct flow pattern that featured two jet streams was observed in the P_B__2_ panel of the bileaflet valve. The jet streams were located at the commissures near the conduit wall. The unique opening behavior combined with the fast opening of the bileaflet valve contributes to this flow phenomenon.

At the peak (*t*_2_), both prostheses have reached the fully open configuration. Although the maximum velocities in the conduits of both prostheses are identical, the different design of prostheses contributes to the differences in velocity and RSS distribution in the conduit. In the bileaflet valve, the jet stream formed a sharp parabola profile with a flat top immediately after the valve in the P_B__1_ panel due to the flow separation. Then, the high-velocity jet reattached at 20 mm downstream the valve tip, which resulted in a square-wave-like velocity profile. In contrast, the high-speed jet has dominated the entire conduit at the level of the commissures in the P_B__2_ panel and developed into a sharp parabola profile at 20 mm downstream the tip of leaflets. One of the major causes of the different velocity distributions in the orthogonal panels is the non-symmetrical flow separation-induced non-circular geometry of the bileaflet valve during the opening. In the trileaflet valve, the reattachment of flow was observed at 40 mm beyond the tip of leaflets.

In the deceleration phase (*t*_3_), a vortex rotating in a clockwise direction near the level of the commissures were observed in all the cases, which is induced by the small jet in the central of conduits.

#### RSS Distributions

It has been well recognized that the RSS level in the vicinity of the valves plays a vital role in the development of thrombosis and valve degradation (Stein et al., 1982; Wheatley et al., 2001; Leo et al., 2006). One of the major mechanobiology mechanisms involved in this progress is the hemolysis and blood damage that occurred in high RSS environments (Yousefi et al., 2017). However, the RSS threshold for hemolysis and blood damage varies, whose range could be between 100 and 5000 Pa (Leo et al., 2006; Jhun et al., 2018). In the aortic site, the peak RSS magnitude during systolic typically ranges from 100 to 450 Pa (Leo et al., 2006).

The results from the current study showed that the highest RSS occurs at the ejection peak for each valve, at which point the streak-like high RSS regions appeared adjacent to the leaflets and developed along the central jet boundary. Similar RSS distribution patterns downstream the bioprosthetic and polymeric aortic valves have been reported by several *in vitro* studies under aortic conditions (Leo et al., 2006; Yousefi et al., 2017; Davis, 2018), and the RSS magnitude levels of both valves are within the physiological range under pulmonary conditions (Stein et al., 1982).

Despite the fact that the maximum RSS of the bileaflet valve is almost identical to that of the trileaflet valve, the bileaflet prosthesis presents a higher overall RSS level in the downstream conduit. This could be attributed to the high leaflet profile of the bileaflet design (Bark et al., 2016).

### Limitations

A major limitation of the current study is that only two valves 25 mm in size were investigated. *In vitro* studies that take the different valve sizes into consideration would be conducted to provide a more comprehensive understanding of the impacts of prosthesis design on the hemodynamic characteristics. In addition, a systemic study on the impacts of variations that might occur in the clinical preparation of the valves should be conducted before their clinical applications.

## Conclusion

The performances of bileaflet and trileaflet ePTFE prosthetic valves were *in vitro* investigated under pulmonary flow conditions in this study. Despite the fact that both of the valves showed excellent hemodynamic performances, the bileaflet design is proved to perform better in terms of the faster opening and closing, lower trans-valvular pressure gradients, smaller RF, larger EOA, and smaller energy loss. Further *in vitro* and *in vivo* investigations of the relation between the potential risk of prosthetic valve dysfunction and higher RSS in the bileaflet design should be conducted.

## Data Availability Statement

All datasets generated for this study are included in the article/supplementary material.

## Author Contributions

JY, MN, and GZ contributed to the conception and design of this study. GZ conducted the experiments and wrote the first draft of the manuscript. YW contributed to the data analysis and visualization. QY and LC contributed to the data interpretation and commented on the manuscript. All authors contributed to manuscript revision, read, and approved the submitted version.

## Conflict of Interest

The authors declare that the research was conducted in the absence of any commercial or financial relationships that could be construed as a potential conflict of interest.
